# Manual versus machine: How accurately does the Medical Text Indexer (MTI) classify different document types into disease areas?

**DOI:** 10.1371/journal.pone.0297526

**Published:** 2024-03-13

**Authors:** Duncan A. Q. Moore, Ohid Yaqub, Bhaven N. Sampat

**Affiliations:** 1 SPRU (Science Policy Research Unit), University of Sussex, Brighton, United Kingdom; 2 School of Public Affairs, Arizona State University, Tempe, Arizona, United States of America; Maynooth University, IRELAND

## Abstract

The Medical Subject Headings (MeSH) thesaurus is a controlled vocabulary developed by the U.S. National Library of Medicine (NLM) for classifying journal articles. It is increasingly used by researchers studying medical innovation to classify text into disease areas and other categories. Although this process was once manual, human indexers are now assisted by algorithms that automate some of the indexing process. NLM has made one of their algorithms, the Medical Text Indexer (MTI), available to researchers. MTI can be used to easily assign MeSH descriptors to arbitrary text, including from document types other than publications. However, the reliability of extending MTI to other document types has not been studied directly. To assess this, we collected text from grants, patents, and drug indications, and compared MTI’s classification to expert manual classification of the same documents. We examined MTI’s recall (how often correct terms were identified) and found that MTI identified 78% of expert-classified MeSH descriptors for grants, 78% for patents, and 86% for drug indications. This high recall could be driven merely by excess suggestions (at an extreme, all diseases being assigned to a piece of text); therefore, we also examined precision (how often identified terms were correct) and found that most MTI outputs were also identified by expert manual classification: precision was 53% for grant text, 73% for patent text, and 64% for drug indications. Additionally, we found that recall and precision could be improved by (i) utilizing ranking scores provided by MTI, (ii) excluding long documents, and (iii) aggregating to higher MeSH categories. For simply detecting the presence of any disease, MTI showed > 94% recall and > 87% precision. Our overall assessment is that MTI is a potentially useful tool for researchers wishing to classify texts from a variety of sources into disease areas.

## Introduction

For more than 50 years, the U.S. National Library of Medicine (NLM) has used the Medical Subject Headings (MeSH) thesaurus, a controlled and hierarchical vocabulary, to classify biomedical publications [1–3]. Increasingly, the MeSH descriptors assigned to publications have allowed researchers studying innovation to develop methods to explore a variety of concepts in the science of science, including creativity [4], mobility [5], mentorship [6], specialization [7], research direction changes [8] and research priorities [9].

Experts at NLM assign MeSH categories to nearly 1 million additions to MEDLINE annually [10]. This classification was once manual, but human indexers today are assisted by algorithmic suggestions [11]. In April 2022, NLM transitioned to 100% automatic indexing and human curation of random samples of the literature [12]. NLM has made available to the public an algorithm it uses to assign MeSH categories to text: the Medical Text Indexer (MTI). The availability of MTI allows arbitrary text, beyond that in publications, to be classified into MeSH categories [13].

MTI is potentially useful for innovation researchers, who work with many document types, including patent literature, information from drug and device approvals, clinical trial registrations, grant descriptions, clinical guidelines, research impact statements, and press releases. Assigning MeSH descriptors to this broad range of documents could also allow texts to be mapped along various dimensions relevant to social science and policy, such as the use of animals in research, clinical versus non-clinical research, model organisms used, and research focused on different age groups, genders, or geographic regions. There are also MeSH categories for diseases. Manual classification of text into diseases and other categories can be costly and require domain-specific expertise [14–19]. MTI potentially allows the classification of large numbers of documents at low cost. For example, researchers have used MTI to assess the gender content of patents [17,20] and the basicness or appliedness of grants funded by the U.S. National Institutes of Health (NIH) [21].

However, it remains unclear whether the applicability of MTI, a tool designed to classify publications for NLM indexing purposes, can be reliably extended to classify text from other document types into disease areas. Ideally, the accuracy of MTI’s classification would be measured against expert manual classification; to our knowledge, there is limited validation of this sort.

Therefore, in this paper, we analyze how well MTI classifies documents by disease area. We manually classified text from different types of documents to one or more descriptors in the MeSH vocabulary. We treated the expert manual classifications as ground truth. For each piece of text, we then evaluated how well MTI performed against these expert manual classifications on two dimensions: (i) recall, i.e. the share of manually assigned MeSH descriptors that MTI identified; and (ii) precision, i.e. the share of MTI outputs that were also identified by expert manual classification. We assessed the average precision and recall of MTI across three different types of documents: grants, patents, and drug indications. We also examined how recall and precision were affected by (i) utilizing the ranking scores provided by MTI to restrict its output, (ii) varying the length of the input text, and (iii) aggregating at different levels of the MeSH disease tree.

## Methods

### Data collection

We collected text from NIH-funded grants, drug patents, and drug indications. To assemble the NIH grant data, we downloaded NIH awards from 2010 from NIH RePORTER/ExPORTER [22] and selected a random sample of four grants per institute/center (including the NIH Office of the Director). This yielded 112 grants. We used information from the ExPORTER database to collect titles and abstracts for each grant. For each grant, we concatenated the title and abstract into one text file, and classified each text file manually and via MTI.

For patents, we focused on those associated with marketed drugs. For small-molecule drugs, such patents are listed in a U.S. Food and Drug Administration (FDA) document, *Drug Products with Therapeutic Equivalence Evaluations*, commonly known as the Orange Book [23]. We drew at random 100 drug patents from the 2015 edition of the Orange Book. After obtaining patent numbers, we downloaded the patent’s title, abstract, and claims from the U.S. Patent and Trademark Office bulk data repository [24]. For each patent, we concatenated the title, abstract, and claims text, and classified the resulting text manually and via MTI.

The final data class we considered was drug indication listings. FDA approvals are for specific indications, which typically include diseases; for example, the drug Elmiron (pentosan polysulfate sodium) was approved by the FDA in 1991 and indicated “for the relief of bladder pain or discomfort associated with interstitial cystitis”. Although indications are typically listed in drug approval packages and correspondence, they have recently been compiled in an FDA database [25]. From this list, we selected a random sample of 100 drugs (new molecular entities) first approved between 2010 and 2015 and used their “Approved Use(s)” text to compile our text file. We classified each drug indication text file manually and via MTI.

S1 Appendix provides the grant, patent, and drug indication text we used for classification.

### MeSH tree structure

**Fig 1:**
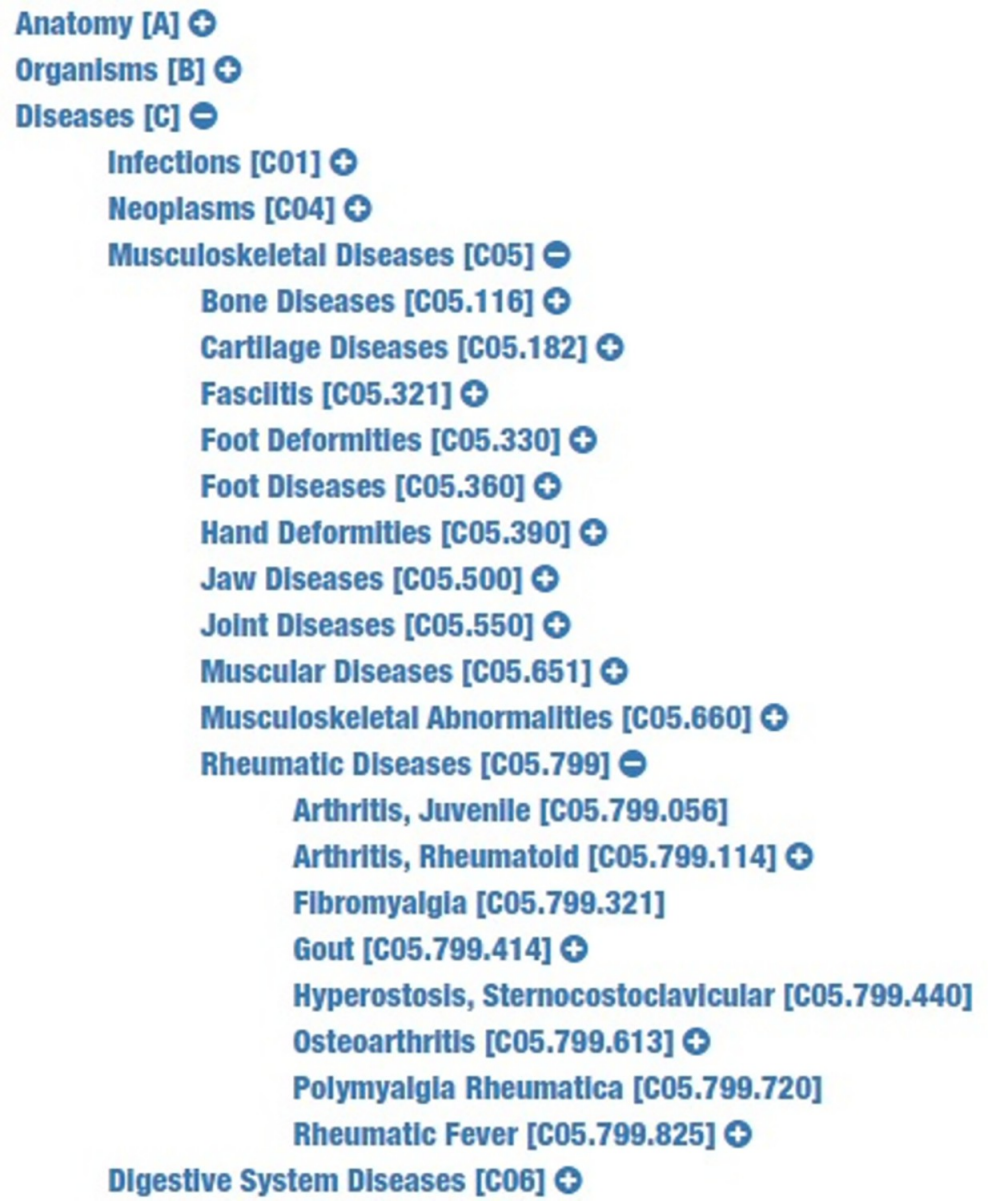
Hierarchical structure of the MeSH tree (an excerpt from the C branch) [26].

MeSH descriptors are organized in a “tree” with 16 main branches, each of which has further sub-branches (Fig 1). The branches include scientific and medical concepts such as anatomy (A), diseases (C), and chemicals and drugs (D), and also broader ideas such as anthropology (I), humanities (K), and disciplines and occupations (H). Because our goal was to classify each input text into diseases in the MeSH vocabulary, we focused on the C branch of the MeSH tree (Fig 1).

The MeSH classification includes hierarchically arranged tree codes, e.g. C05.799.114 for “arthritis, rheumatoid”, which is under the “rheumatic diseases” subbranch (C05.799), which in turn is under the “musculoskeletal diseases” branch (C05). Each code has a descriptor (in this case, “arthritis, rheumatoid”). Each descriptor has a unique code, known as a MeSH Unique ID (UI; not displayed here); as an example, D001172 is the UI for “arthritis, rheumatoid”. But a descriptor/UI may have multiple tree codes; for example, “arthritis, rheumatoid” (D001172) has three other tree codes as well. In addition to being listed twice on the C05 branch (“musculoskeletal diseases”) of the MeSH tree, it is also on the C17 branch (“skin and connective tissue diseases”) and the C20 branch (“immune system diseases”). Accordingly, in our manual classification we focused on identifying the MeSH descriptors associated with different pieces of text and compared them with the MeSH descriptors that MTI returned for the same text.

### Manual classification

One of the authors (DM), with an MBiochem from the University of Oxford and a PhD from the University of Sussex, was the expert classifier. Manual classification sought to assign to texts the most specific MeSH terms possible. We treated expert manual classification as the gold standard in classifying grant, patent, and drug indication text. To test the validity of this assumption, we manually classified the title and abstract text from 100 articles published in 2018 in the general medical journal *The Lancet* into one or more C-branch MeSH terms and compared our classification to the NLM-assigned MeSH terms listed in MEDLINE/PubMed. On average, our manual classification showed 83% recall and 95% precision. This compares well with prior benchmarks [27].

After validating that our manual classification worked well for publications, we manually classified 312 pieces of grant, patent, and drug indication text to the C branch of the MeSH tree. Specifically, after reading the text, we determined the MeSH descriptors for all diseases mentioned in the text.

### MTI classification

We passed each text unit through the MTI batch indexer as input data [28], with the following settings:

- Filtering: default for non-MEDLINE text
- Post processing options: show tree codes, show term unique identifiers, show MeSH DUIs (descriptor UIs)
- Output: just the facts
- Batch-specific options: single-line delimited input with ID

**Fig 2:**
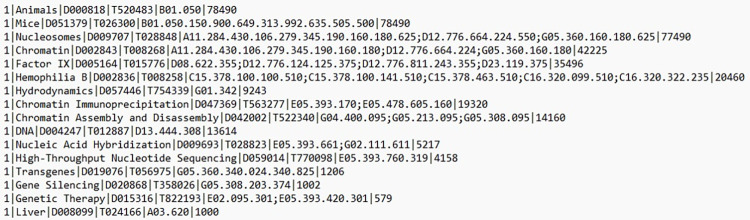
An example of MTI output, using text from a grant description titled “An effort to create factor IX expression vectors with sustained expression”.

MTI outputs MeSH descriptors, their UIs, and all tree branch codes, as well as a ranking score for each term it assigns to the inputted text. The ranking scores indicate “the confidence or strength of belief in the assignment” [29, page 6] and are used to rank the suggested terms for the input text. NLM provides further details on how MTI calculates these scores [29,30]. In some of our analyses, we used these ranking scores to filter MTI suggestions, focusing on the top three, top five, etc. Fig 2 shows an example of raw MTI output from the batch indexer.

Note that in this example, as is common, the MTI output includes many non-disease descriptors that do not appear anywhere on the C branch of the MeSH tree. We excluded these when evaluating MTI’s performance, because the specific focus of our analyses was on the accuracy of MTI’s disease classification as compared with our manual assignment of disease-branch descriptors for the same text. We also excluded supplementary concepts, since these are not part of the main controlled vocabulary. Where there were texts for which both manual classification and MTI returned no disease descriptors, this was counted as an agreement on a lack of disease.

With these data, we calculated MTI’s recall: the share of manually assigned MeSH descriptors that MTI identified for each piece of text. This share would be mechanically high if MTI were liberal with its suggested outputs (at an extreme, assigning all diseases to a piece of text), so we also calculated its precision: the share of MTI outputs that were also identified by expert manual classification. We dropped texts where recall or precision could not be calculated due to a zero denominator. These occurred in two scenarios: for recall, when manual classification assigned no disease but MTI identified disease(s); and, for precision, when manual classification assigned disease(s) but MTI identified none. We then averaged the recall and precision values for all remaining texts within each document type. In this way, we examined the performance of MTI in terms of its mean average recall and precision, for each document class.

Our approach to assessing multi-label classification focused on examples of texts used. Another approach could be to focus on the descriptors being assigned. For each descriptor, recall and precision would be calculated, and averaged over the entire dataset. The descriptor-based approach would show how MTI performance is distributed over the 4,933 possible descriptors (which could be important if, say, there is concern that rarely used descriptors might be more prone to errors). However, in this study, we were interested in potentially extending the use of MTI across document types. So, rather than the descriptor-based approach, we proceeded with the example-based approach that focused its recall and precision measures on the texts and then averaged across datasets. For further discussion of example-based versus label-based classification is available [31,32].

### Exploration of factors affecting recall and precision

MTI misclassifications can smooth over after aggregation. For example, consider a piece of text about prostatic neoplasms and categorized manually as such. If MTI were to classify this as “breast neoplasms”, that would be a mistake. But both “prostatic neoplasms” and “breast neoplasms” are on the C04 (“neoplasms”) branch of the MeSH tree, so at a higher level of aggregation, the classification would be correct. To quantify how aggregation influences performance, we additionally examined recall and precision at the aggregated level. We aggregated the MTI and manual outputs to the second hierarchical level of the MeSH tree (subcategories C01, C04, C05, etc.), which reduced the number of possible classifications from 4,933 to 21. We also aggregated to the first hierarchical level (C or non-C branch); this allowed us to compute agreement between MTI and manual classification on whether a text was assigned any disease, i.e. a binary classification of no-disease or any-disease.

One difficulty with aggregation is an issue raised earlier, that a MeSH descriptor can map to multiple branches of the MeSH tree. We opted to allow a single MeSH descriptor to map to multiple branches of the MeSH tree but only once per branch. For “arthritis, rheumatoid”, which has four locations in the MeSH tree, aggregation would convert its MeSH descriptor to three tree codes, corresponding to the C05, C17, and C20 branches, because it appears in C05 twice and in C17 and C20 once.

We examined the potential for using MTI’s ranking scores. This is because MTI’s precision could be improved if we restricted its outputs to those with high-ranking scores (indicating a high level of confidence in the MeSH term assignments). However, restricting its outputs in this way may also adversely affect its recall performance. Therefore, we examined this possible trade-off with thresholding, in terms of how MTI’s recall and precision would be affected if we restricted MTI outputs to its top two, top three, top five, etc.

Finally, we explored text length and number of disease categories. Some grant descriptions, patent texts, and drug indications are longer than others and/or feature multiple disease categories. This variation might affect performance. However, the direction in which length may affect performance was not clear *ex ante*. Fewer words may be more challenging for MTI because the algorithm has less information to work with, but conversely, feeding it more text than necessary may introduce additional potential sources of error. Therefore, we examined how MTI performance varied when texts were relatively short (below median length) or long (above median length) for each document type.

## Results

We calculated recall (Table 1) and precision (Table 2) for every text processed by MTI and then averaged these for each document type. We also calculated recall and precision after aggregating MTI outputs to the second hierarchical level of the MeSH tree (subcategories C01, C04, C05, etc.) and the first hierarchical level (C or non-C branch), and after restricting them to those in the top three scoring MeSH descriptors for a text.

**Table 1:** Recall averages (shares of manually assigned MeSH descriptors correctly identified by MTI).

Document class	Recall (%)	Recall, from top three MTI outputs (%)	Recall, aggregated to second hierarchical level (%)	Recall, aggregated to first hierarchical level (%)
Grant descriptions	77.8	76.3	90.1	98.0
Patent text	77.6	71.7	86.1	94.3
Drug indications	85.5	85.5	97.0	97.9

**Table 2:** Precision averages (shares of MTI MeSH descriptors that were correctly suggested).

Document class	Precision (%)	Precision, from top three MTI outputs (%)	Precision, aggregated to second hierarchical level (%)	Precision, aggregated to first hierarchical level (%)
Grant descriptions	53.2	54.4	68.4	88.2
Patent text	72.6	74.1	80.0	87.4
Drug indications	64.2	65.6	81.2	93.9

With > 53% precision across the board (Table 2), MTI was often correct, although it did output some incorrect diseases. MTI had superior precision when working with patent text (72%) compared with drug indications (64%) or grant descriptions (53%). Restricting to the three highest-scoring outputs, MTI showed similar precision. Aggregating to the second hierarchical level improved precision by 7–17 percentage points. Aggregating to the first hierarchical level, to consider the presence of any disease descriptor whatsoever in a text, precision increased to 87% or greater.

For grants and patents, MTI had a recall of 78%. For drug indications, MTI seemed to perform even better, with a recall of 86%. When restricting its output to the three highest-scoring MeSH descriptors for each piece of text, MTI still showed similar recall. When aggregating to the second hierarchical level, MTI recall increased to 86% or greater. When aggregating yet further to the first hierarchical level, to consider the presence of any disease descriptor whatsoever in a text, these recall figures again increased to 94% or greater.

**Fig 3:**
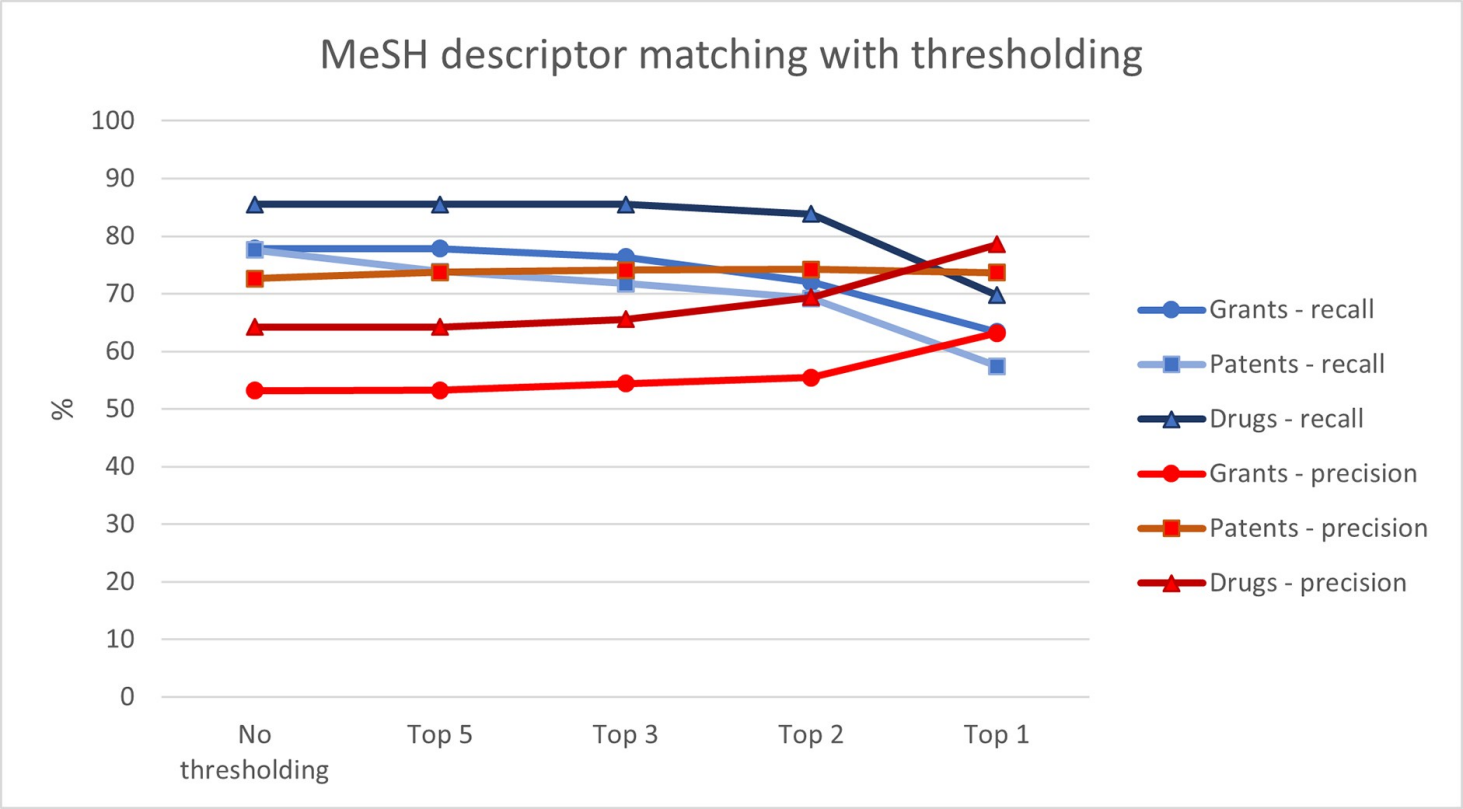
Recall and precision averages with increasingly stringent thresholding by score.

Fig 3 shows MTI’s recall and precision performance when restricted to a varying number of outputs. Its recall was > 77%, even when restricted to its top three ranked MeSH descriptors. If restricted to only the top one output, recall dropped sharply, with few gains in precision. MTI’s recall was strongest for drug indications, and its precision was strongest for patent text.

Fig 3 also shows that MTI’s recall was not simply mechanically high as a result of assigning an excessive number of disease labels to a piece of text. In fact, its precision was > 53%, indicating that more than half of its outputs were also identified by expert manual classification. However, with two or more MTI outputs, its precision was slightly lower than its recall performance. With no thresholding, MTI’s recall was slightly better than its precision for all document types.

We found that the majority of our texts were manually assigned zero categories or one category only: 83% of grant descriptions, 68% of patent texts, and 77% of drug indications. When we restricted the input to only these texts (i.e. excluding multiple disease category texts), MTI performed similarly, often identifying the manually assigned MeSH descriptor: on average, 83% for grant descriptions, 84% for patent texts, and 89% for drug indications. We found the average share of MTI outputs that were also identified by manual classification: 53% for grant descriptions, 69% for patent texts, and 62% for drug indications. Selecting for these texts produced moderate improvements in recall, with only slight decreases in precision.

MTI showed better recall and precision with texts that were short (below the median length) relative to other texts in the same document types (Table 3).

**Table 3:** Recall and precision averages for texts of different lengths.

Document type	Recall (%)	Precision (%)
Long grant descriptions (> 2738 characters)	77.7	49.3
Short grant descriptions	78.0	57.1
Long patent text (> 4070 characters)	73.5	67.1
Short patent text	81.7	78.2
Long drug indications (> 310 characters)	81.3	58.1
Short drug indications	89.7	70.1

Performance gains were most noticeable for patent text and drug indications, with improvements of 8–12 percentage points in terms of both recall and precision. For grant descriptions, recall was largely unaffected by relative text length, although precision still improved with shorter grant descriptions, by nearly 8 percentage points. This indicates that MTI’s recall and precision falls for input text that is unusually long for its document class and suggests that the longest of texts for a given document type may be particularly prone to errors. Moreover, it suggests that performance differences between document types arise at least partly because these document types tend to differ in text length. The reason why text length should affect performance in this way remains to be clarified.

To offer an illustrative use-case for MTI, we singled out texts assigned with C04 (“neoplasms”) MeSH terms, and any MeSH terms under the C04 branch. We asked what share of grants, patents, and drugs that have manually assigned ‘cancer’ terms does MTI call cancer, and vice versa, by using the C04 “neoplasms” MeSH term and all terms under it. For grant descriptions, we found 15 grants that we called cancer, of which MTI called 14 cancer; and we found 22 grants that MTI called cancer, of which 14 were actually cancer. For patent texts, we found eight patents that we called cancer, of which MTI called all cancer; and we found eight patents MTI called cancer, of which six were actually cancer. For drug indications, we found 20 drugs that we called cancer, of which MTI called all cancer; and we found 24 drugs MTI called cancer, of which 20 were actually cancer.

S1 Appendix are provided for the replication of results described in this section.

## Discussion

In this study, we evaluated the accuracy of MTI in categorizing various types of texts into disease areas, by comparing the MeSH descriptors yielded by MTI with those identified by expert manual classification of the same texts. We found that MTI’s recall was 78% for grant descriptions and patent text, and 86% for drug indications (Table 1). Furthermore, we confirmed that this strong recall performance was not simply due to MTI being liberal with its outputs, because its precision was 53% for grant descriptions, 73% for patent text, and 64% for drug indications (Table 2). Additionally, we found that MTI’s recall and precision could be improved further by aggregating to categories at the second and first hierarchical levels of the MeSH tree, and by restricting the input text to shorter documents.

We found that MTI did suggest some diseases incorrectly, in addition to its correct suggestions. MTI’s precision was lower than its recall for all document types. Making use of the ranking scores output alongside the MeSH descriptors is one way of exercising greater caution where particularly clean data is desired. We found that restricting to only the top one or two highest-scoring outputs resulted in a substantial improvement in precision (up to 14 percentage points) (Fig 3). In the context of drug indications, MTI precision could be improved by ensuring that contraindications are removed from the input text. This can be illustrated by the following excerpt from the “Approved Use(s)” text for the drug Invokana: “Invokana (canagliflozin) is indicated as an adjunct to diet and exercise to improve glycaemic control in adults with type 2 diabetes mellitus. Limitation of Use: Invokana is not recommended in patients with type 1 diabetes mellitus or for the treatment of diabetic ketoacidosis.” MTI identified the drug indication of type 2 diabetes but also incorrectly identified the contraindications of diabetic ketoacidosis and type 1 diabetes. For this document type, researchers may wish to screen for common phrases such as “limitations of use” or “not recommended for” to prevent contraindications from being presented to MTI.

Our results complement prior efforts to explore the accuracy of MTI. A previous study evaluated the assignment of the check tags “male” and “female” to patent text and compared MTI outputs with expert manual assignment [17]. Check tags (so called because historically they were available to indexers on pre-printed forms as a box to check) are MeSH categories that cover frequently occurring concepts, and they are routinely added for each article during the indexing process for MEDLINE. The results of that study suggested that MTI performs well when assigning these specific check tags to patent text [17]. Our results suggest similar recall and precision performance at the aggregated disease category level when assigning descriptors to patent texts, grant descriptions, and drug indications. In addition to showing similar performance in a wider range of texts, our results are also notable because disease categories occur less frequently than check tags. Recall and precision performance might have been expected to be high only for frequently occurring categories (check tags), but we found that this is not the case. That said, it should be noted that by focusing on patents associated with drugs, rather than including all biomedical patents, we may be overstating confidence in MTI (or in humans). Non-drug biomedical patents (or upstream ones) may be harder to classify, both manually and by machine.

MTI performs considerably better when presented with texts that are short relative to other texts in a given document type (Table 3), missing fewer diseases and suggesting fewer incorrect diseases. We observed performance gains across all three document types, most noticeably in patent text and drug indications. Although the reasons for these differences are not yet clear, we could see that precision was more adversely affected than recall, suggesting that longer texts seem to generate more noise than signal when determining disease areas. Researchers using MTI may wish to exclude certain texts (e.g. the top 5% of a document type by text length) when checking the robustness of their results, particularly if they are working with patent texts.

Another method that improved MTI’s performance was aggregation to the second hierarchical level. An example of this was a grant on type 2 diabetes, for which MTI produced the incorrect codes for hypertension, cardiovascular disease, and heart disease, all of which are solely in the C14 category. In this case, aggregation converted three errors into only one error, and we can see that the performance improvement occurs through approximation. Where approximate disease categories are sufficient, aggregation can be potentially useful for researchers using MTI.

If all that is required of MTI is simply to detect the presence or absence of any disease whatsoever in a text, then MTI performs this task assuredly (i.e. aggregating to the first hierarchical level). For binary classification into no-disease or any-disease, MTI showed > 83% recall and > 87% precision. We think this is likely to be close to human inter-rater subjectivity scores.

However, when considering the use of aggregation to improve MTI performance, there are at least two notes of caution to sound. First, aggregation may yield inconsistent results by disease area and counting method. This is because some MeSH descriptors repeat across and within branches of the MeSH tree more than others. For example, the MeSH term “glomerulonephritis, membranoproliferative” has tree codes on the third, seventh, eighth, and ninth hierarchical levels, spanning two sub-branches (C12 and C20). Faced with this repetition for some disease areas, as highlighted in the Methods, aggregation requires researchers to make difficult choices that may not be obvious when initially considering aggregation, such as how many times to count a descriptor that appears in multiple branches, or whether to count only its first mention in the hierarchy or more than one mention within a branch. We opted to examine only very coarse aggregation or none at all. Attempts to aggregate to finer levels, in between the second and deepest levels, may also yield results that differ by disease area. Recall and precision performance may therefore vary depending on the underlying population of diseases in the input texts and how the aggregation is counted.

Second, aggregation may result in different kinds of approximations. In some instances, aggregating up one level may combine very similar disease areas (e.g. folding “prostatic neoplasms, castration-resistant” into “prostatic neoplasms”), whereas in others, it may combine areas that are much more distinct (e.g. “bacterial endocarditis”, “cardiovascular syphilis”, and “cardiovascular tuberculosis” would be combined into one category). This problem persists at deeper levels of the hierarchy, where aggregation can yield results as specific as “glomerulonephritis, membranoproliferative” or as broad as “bacterial infections and mycoses”, both located at the third hierarchical level. We view these as issues with the MeSH tree rather than with MTI, and they are likely to surface in most classification systems when examined closely [33].

Our qualitative review suggested that, where MTI produced errors, many of the input texts were inherently difficult to categorize, whether by human or machine [33]. Multiple categories are often needed to capture what a text is about, leading to some diseases being missed by MTI, which affected its recall; and it may well be that certain diseases require qualification with non-disease terms before they warrant inclusion, leading to some incorrect diseases being generated by MTI, which affected its precision. It is possible to envisage similar situations faced by clinicians in disagreement on points of diagnosis [34].

Despite these challenges, MTI’s recall and precision showed that it was able to reliably identify diseases, with few errors, in grant descriptions, patent texts, and drug indications. Overall, our evaluation suggests that MTI is a potentially useful tool for researchers wishing to categorize texts from a variety of sources into disease categories.

## Supporting information

S1 Appendix (ZIP)
