# Supplementary figures and images for: Manual versus machine: How accurately does the Medical Text Indexer (MTI) classify different document types into disease areas?
Source: PLoS One. 2024 Mar 13;19(3):e0297526. doi: 10.1371/journal.pone.0297526 (PMC10936797; doi:10.1371/journal.pone.0297526)

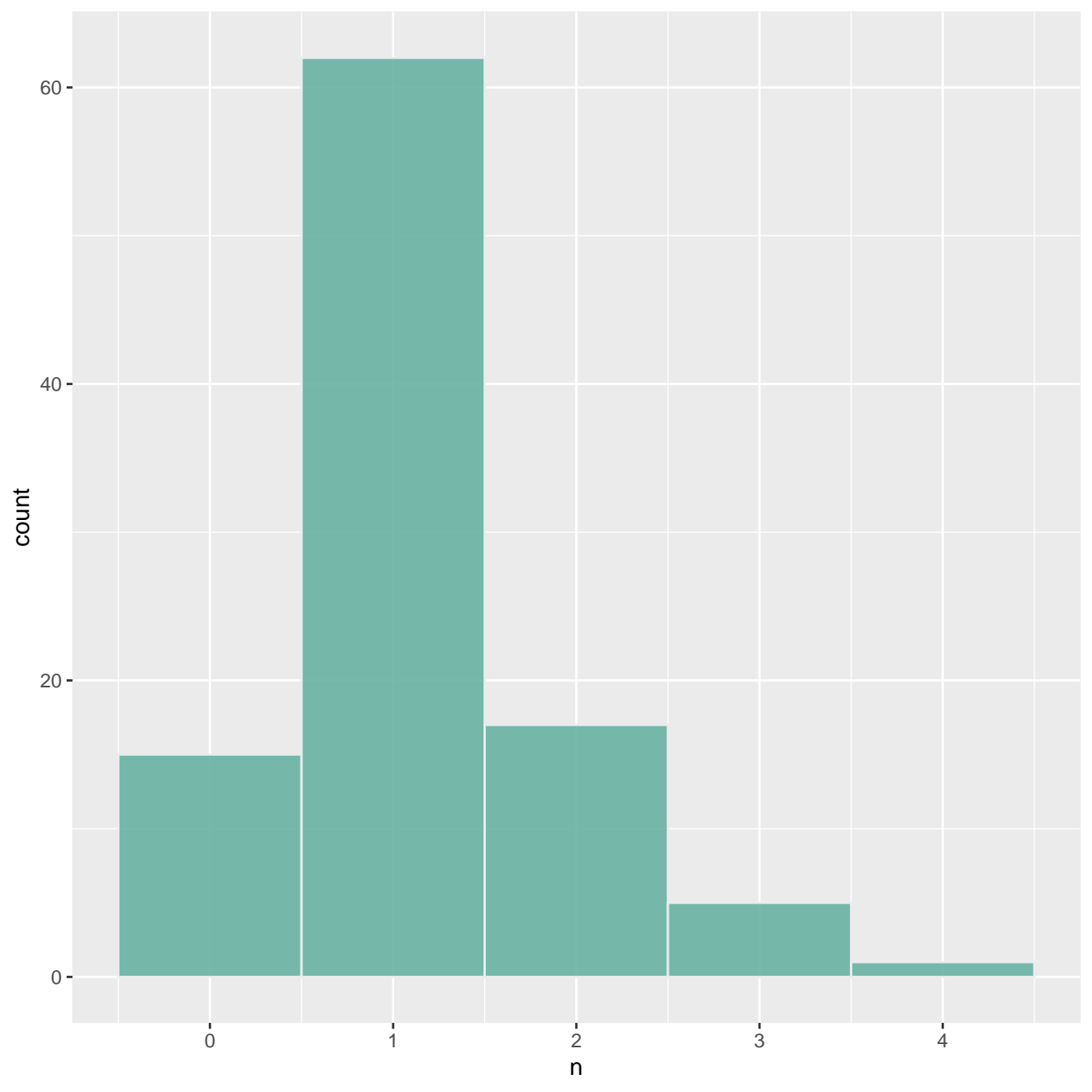

Supplement: S1 Appendix — (ZIP) [file pone.0297526.s001.zip › histograms_no_of_codes/hist_man_d.pdf]

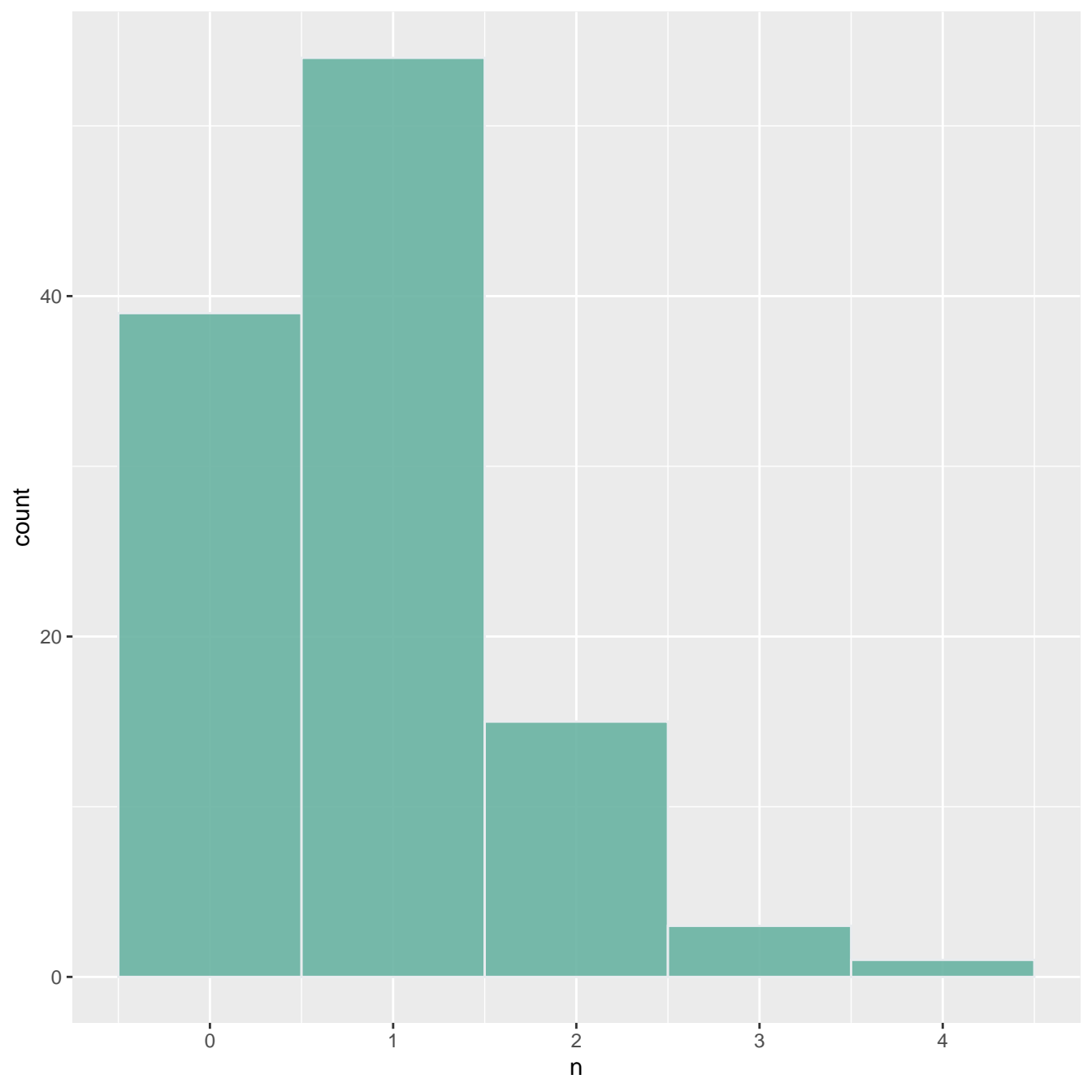

Supplement: S1 Appendix — (ZIP) [file pone.0297526.s001.zip › histograms_no_of_codes/hist_man_g.pdf]

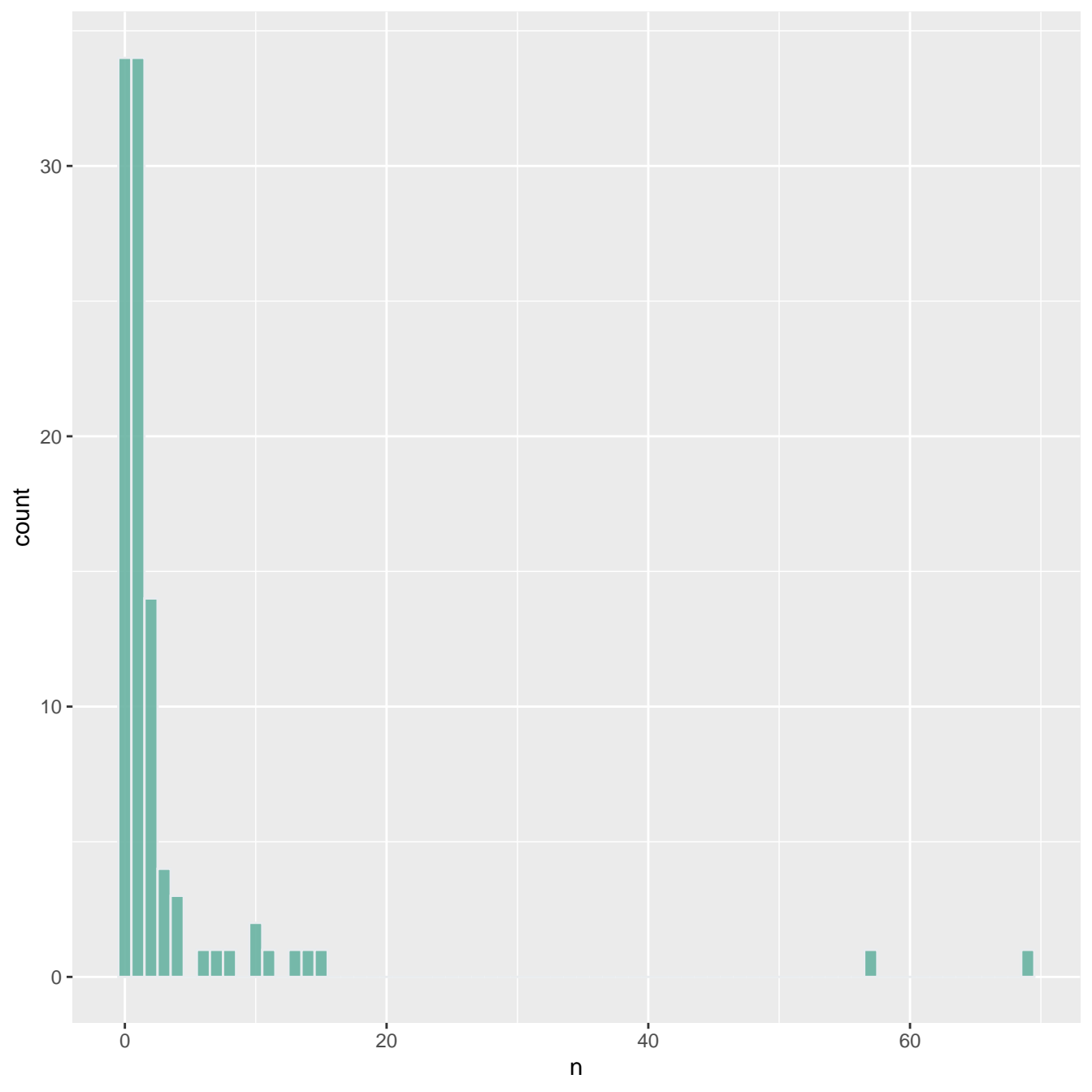

Supplement: S1 Appendix — (ZIP) [file pone.0297526.s001.zip › histograms_no_of_codes/hist_man_pt.pdf]

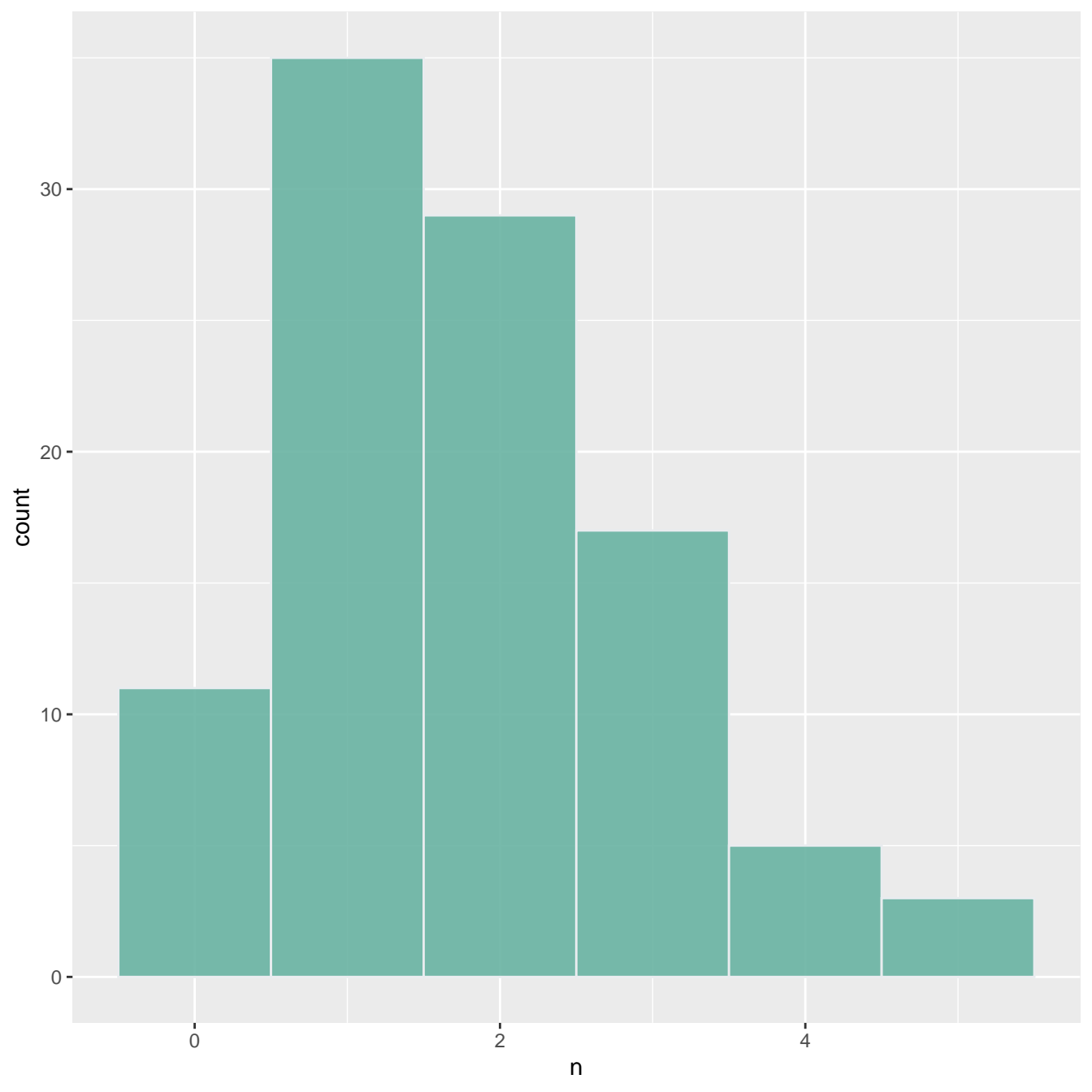

Supplement: S1 Appendix — (ZIP) [file pone.0297526.s001.zip › histograms_no_of_codes/hist_mti_d.pdf]

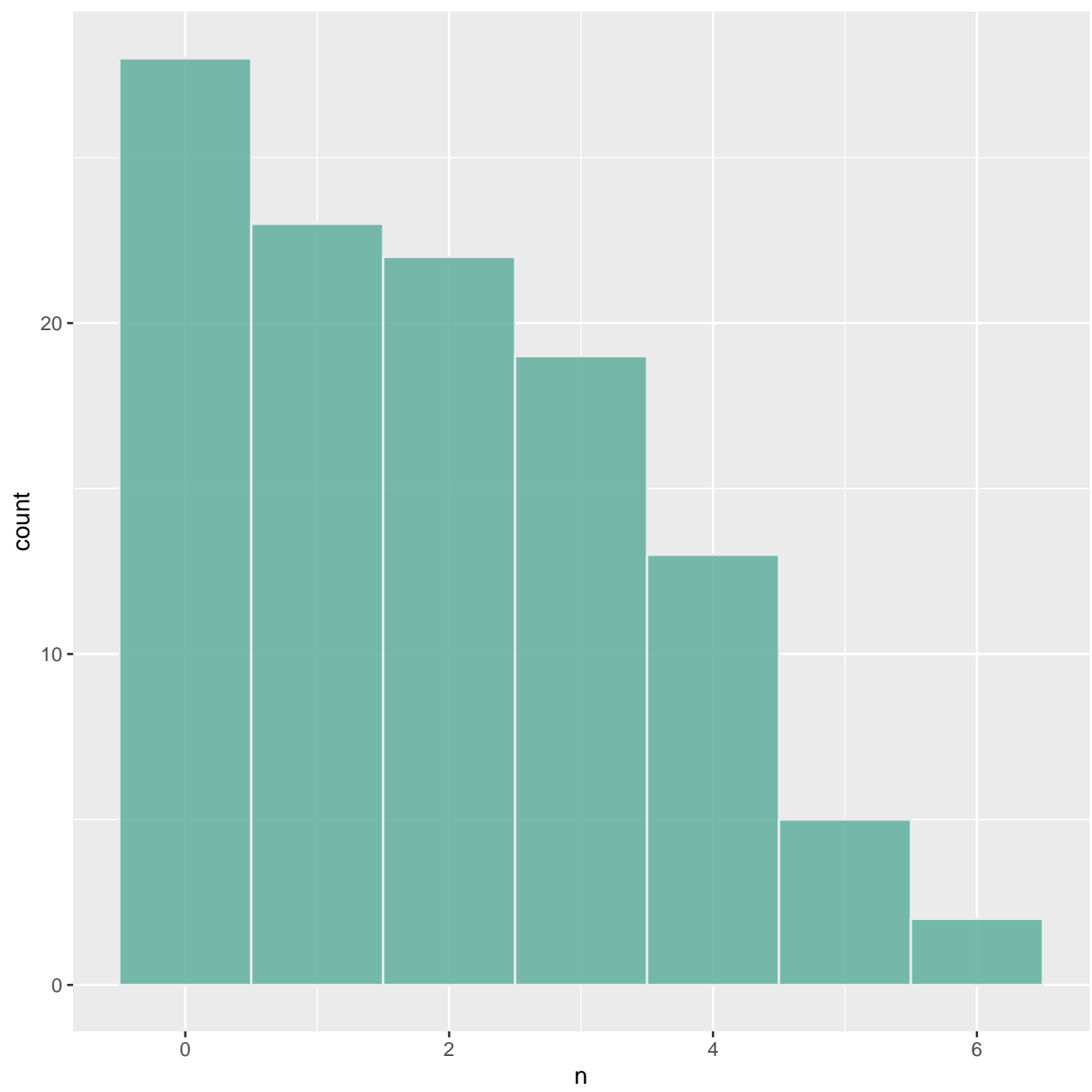

Supplement: S1 Appendix — (ZIP) [file pone.0297526.s001.zip › histograms_no_of_codes/hist_mti_g.pdf]

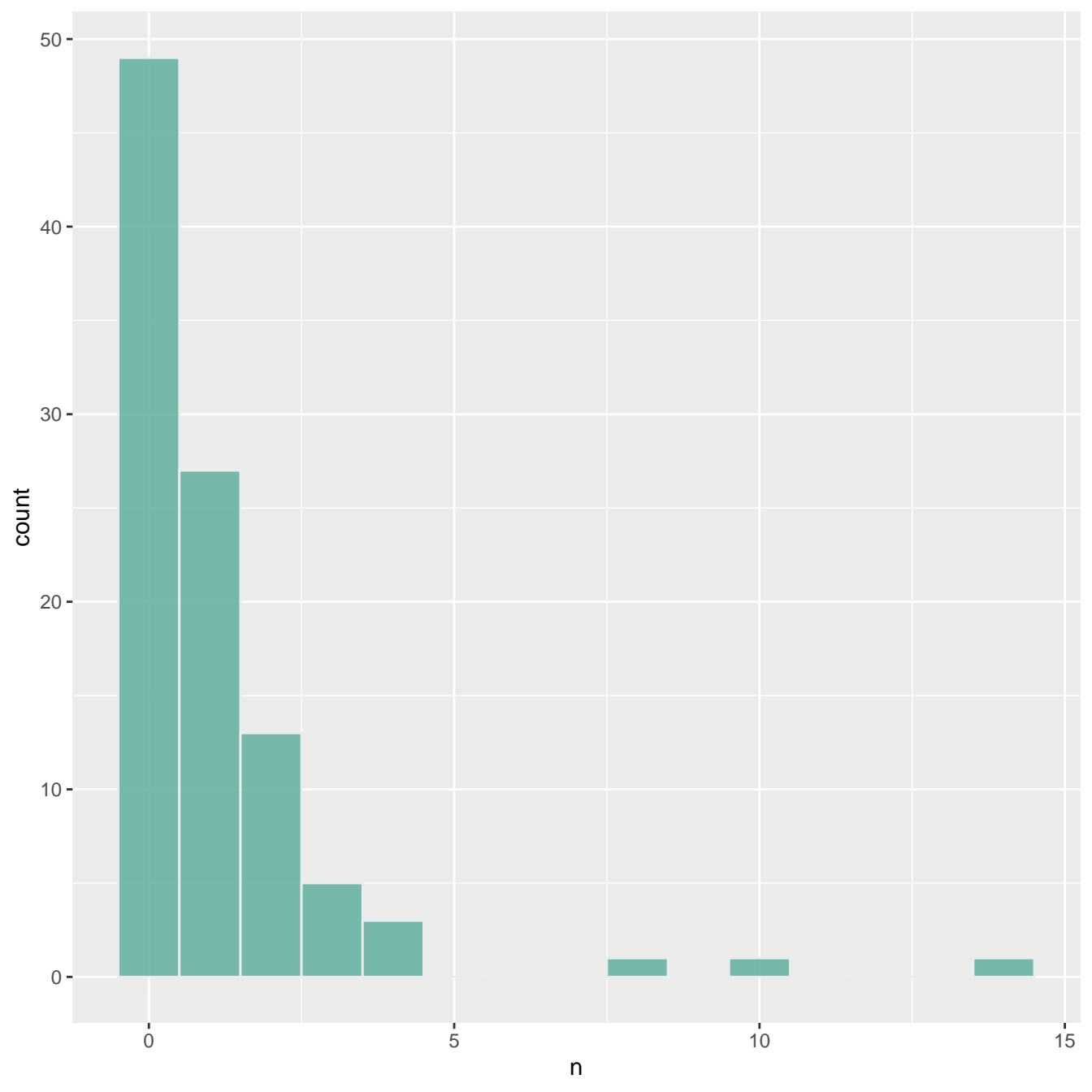

Supplement: S1 Appendix — (ZIP) [file pone.0297526.s001.zip › histograms_no_of_codes/hist_mti_pt.pdf]
